# Public-private settlement and hospital mortality per sources of payment

**DOI:** 10.1590/S1518-8787.2016050006330

**Published:** 2016-07-12

**Authors:** Juliana Pires Machado, Mônica Martins, Iuri da Costa Leite

**Affiliations:** I Diretoria de Desenvolvimento Setorial. Agência Nacional de Saúde Suplementar. Rio de Janeiro, RJ, Brasil; II Departamento de Administração e Planejamento em Saúde. Escola Nacional de Saúde Pública. Fundação Oswaldo Cruz. Rio de Janeiro, RJ, Brasil; III Departamento de Epidemiologia e Métodos Quantitativos em Saúde. Escola Nacional de Saúde Pública. Fundação Oswaldo Cruz. Rio de Janeiro, RJ, Brasil

**Keywords:** Hospital Mortality, Private Health Care Coverage, Public-Private Sector Partnerships, Unified Health System, Outcome and Process Assessment (Health Care)

## Abstract

**OBJECTIVE:**

To analyze if the adjusted hospital mortality varies according to source of payment of hospital admissions, legal nature, and financing settlement of hospitals.

**METHODS:**

Cros-ssectional study with information source in administrative databases. Specific hospital admission reasons were selected considering the volume of hospital admissions and the list of quality indicators proposed by the North-American Agency for Healthcare Research and Quality (AHRQ). Were analyzed 852,864 hospital admissions of adults, occurred in 789 hospitals between 2008 and 2010, in Sao Paulo and Rio Grande do Sul, applying multilevel logistic regression.

**RESULTS:**

At hospital admission level, showed higher chances of death male patients in more advanced age groups, with comorbidity, who used intensive care unit, and had the Brazilian Unified Health System as source of payment. At the level of hospitals, in those located in the mean of the distribution, the adjusted probability of death in hospital admissions financed by plan or private was 5.0%, against 9.0% when reimbursed by the Brazilian Unified Health System. This probability increased in hospital admissions financed by the Brazilian Unified Health System in hospitals to two standard deviations above the mean, reaching 29.0%.

**CONCLUSIONS:**

In addition to structural characteristics of the hospitals and the profile of the patients, interventions aimed at improving care should also consider the coverage of the population by health plans, the network shared between beneficiaries of plans and users of the Brazilian Unified Health System, the standard of care to the various sources of payment by hospitals and, most importantly, how these factors influence the clinical performance.

## RESUMO

**OBJETIVO:**

Analisar se a mortalidade hospitalar ajustada varia segundo fonte de pagamento das internações, natureza jurídica e arranjo de financiamento dos hospitais.

**MÉTODOS:**

Estudo observacional transversal com fonte de informações em bases de dados administrativos. Motivos de internação específicos foram selecionados considerando o volume de internações e a lista de indicadores de qualidade propostos pela agência norte-americana de pesquisa em saúde e qualidade (AHRQ). Foram analisadas 852.864 internações em adultos, ocorridas em 789 hospitais entre 2008 e 2010, em São Paulo e Rio Grande do Sul, aplicando regressão logística multinível.

**RESULTADOS:**

No nível da internação, apresentaram maiores chances de óbito pacientes do sexo masculino, em faixas etárias mais avançadas, com comorbidade, que utilizaram unidade de terapia intensiva, e tinham o Sistema Único de Saúde como fonte de pagamento. No nível dos hospitais, naqueles situados na média da distribuição, a probabilidade de morte ajustada nas internações financiadas por plano ou particular foi de 5,0%, contra 9,0% quando reembolsadas pelo Sistema Único de Saúde. Essa probabilidade aumentou nas internações financiadas pelo Sistema Único de Saúde em hospitais a dois desvios padrão acima da média, passando para 29,0%.

**CONCLUSÕES:**

Além das características estruturais dos hospitais e do perfil dos pacientes, intervenções voltadas para a melhoria do cuidado deveriam considerar também a cobertura da população por planos de saúde, a rede compartilhada entre beneficiários de planos e usuários do Sistema Único de Saúde, o padrão de atendimento às diversas fontes de pagamento pelos hospitais e, fundamentalmente, como esses fatores influenciam o desempenho clínico.

## INTRODUCTION

The Brazilian health system presents peculiarities related to the way the public-private settlement results in health care. Hospital care is mainly carried out by private entities, which can simultaneously attended patients funded by the Brazilian Unified Health System (SUS) and by private health plans, producing complex financing settlements and assistance networks that make the regulation of the system difficult.

The interaction between SUS and health plans has been considered a factor that produces inequality in the access, use and, quality of health services^22^. In addition, the public-private competition for the same network of providers, without separation of the clientele served, raises problems in the organization of care and impairs the relationship of complementarity expected for the system^2^.

Hospital mortality has been one of the most exploited indicators to measure hospital quality for representing an unequivocal result, a global measure of the care process, and also for its availability in the administrative databases^5,6,19^. Hospital mortality must be adjusted for the risk of the patients, to enable more reliable comparisons of providers. Traditionally, these adjustments include, in regression models, attributes of patients expressing degrees of risk of death^6,13,16,23^. In addition to the characteristics of the patients, the structure and functioning of hospitals may also affect hospital mortality. Therefore, the variation in hospital mortality can indicate problems in the quality of the care provided by hospitals. In this sense, to measure different explanatory factors involved is crucial in this approach, although it is not trivial.

In addition, complicating this type of analysis, there is the fact that patients in the same hospital have more similarity between themselves than with patients in different hospitals, generating dependency between observations within each hospital. To deal with this dependency, multilevel regression models have been used in some studies^21^.

The relationship between hospital mortality and structural and organizational characteristics of hospitals has received great attention in national and international literature^4^, with emphasis on the analysis of public or private nature^2,8^ and on source of financing for hospital admissions^25,27^. In Brazil, studies developed, in general, are restricted to certain geographical areas^12,15^. This is mainly due to the unavailability of secondary data with acceptable coverage and quality of fulfillment to the whole Brazilian territory^a^.

This study aimed to analyze if adjusted hospital mortality varies according to source of payment of hospital admissions, legal nature, and financing settlement of hospitals.

## METHODS

### Study universe

Were studied hospital admissions carried out between 2008 and 2010 in hospitals in Sao Paulo and Rio Grande do Sul, with better coverage and quality of fulfillment of Hospital Admissions Communication (CIH)^a^.

Of 39,419,539 hospital admissions recorded during this period, 7,385,323 were selected for patients aged between 18 and 99 years, admitted less than 30 days (acute), not transferred, in the specialties general surgery or internal medicine, whose procedures and diagnoses were unrelated to obstetrics, not occurring in hospitals with at least one hospital admission/day.

From these 7,385,323 hospital admissions, the study universe integrated the 852,864 regarding four causes with more admissions and deaths in Brazil, among the seven proposals for quality indicators for the Agency for Healthcare Research and Quality – AHRQ)^b^. They are: acute myocardial infarction – AMI (CID 21); congestive heart failure – CHF (CID I50, I11, I13); cerebrovascular accident – CVA (CID I60 I61 I62 CID, I63, I64); and pneumonia (CID J13, J14, J15, J16, J18).

The use of these causes is based on greater causal validity of hospital mortality because of the care applied to such diagnoses, with evidences indicating relationship between process of care and survival of the patients^b^. The same causes are used in monitoring and evaluations of quality in other countries, including comparisons between them^5,19^.

### Databases

Were used administrative databases provided by the Department of Informatics of SUS (DATASUS) and by the National Health Agency (ANS), both linked to the Brazilian Ministry of Health.

For the SUS-financed admissions, data are from the System of Hospital Information of SUS (SIH/DATASUS); for the admissions whose source of payment were private or of health plan, data have as a source the CIH/DATASUS. For both cases are publicly available on the DATASUS website files with individualized data (however de-identified) of patients.

Data on the structure of public and private hospital network came from the National Registry of Health Establishments (CNES/DATASUS), also obtained in the website. Further data on private network have been obtained from the System of Health Plans Registry (RPS/ANS), upon request of information to the ANS.

### Analyses

The dependent variable was the occurrence of in-hospital death. Data analysis was carried out in two steps: (1) risk adjustment model; (2) analysis of characteristics of the care process and the structure of hospitals.

The risk adjustment model included the following variables: sex, age, primary diagnosis, presence of comorbidity, Charlson Comorbidity Index (CCI), and clinical conditions of Elixhauser not covered by CCI. Several models were tested from the base model composed by age and sex, then including other variables. The variable type of admission (elective or emergency) has not been tested because it was not fulfilled in the CIH database. Although information about comorbidity are restricted in the sources of information used, the two comorbidity indexes have been used aiming to greater precision of risk adjustment models, qualifying them to represent a degree of information about comorbidity. The algorithm of Quan et al.^16^ was used in the construction of these indexes.

To assess the predictive ability of the models tested, the C-statistics (Receiver Operating Characteristics – ROC curve scores) was used. This test measures the probability predicted of risk of death in a randomly selected patient who died, compared to the probability of risk of death in a randomly selected patient who survived. For this statistics, the value of 0.5 suggests that the model is indifferent to a random chance of predicting death, while 1.0 suggests perfect discrimination; values up to 0.7 are considered of low discrimination, between 0.7 and 0.8, of moderate discrimination, and above 0.8, the model is considered predictive and of high discrimination^1^.

In the second step, variables regarding hospital admission level were: length of stay, type of procedure performed (surgical or clinical), use of Intensive Care Unit (ICU), and source of payment of hospital admission (SUS, health plan, or private). The variables regarding hospital level included were: legal nature, financing settlement, capacity, and educational activity.

As independent central variables in this study, it has been privileged: source of payment of the hospital admission, legal nature (public, for-profit private and non-profit private), and financing settlement (only SUS; SUS, health plans, and private; health plan and private) of the hospital.

The variable financing settlement is one of the types of institutional settlements, a term from the field of economics to describe forms of organization between market agents^20^. The financing settlement was built based on registration information and hospital production, each category indicating a combination in the sources of payment of admissions carried out in the hospital: “only SUS” refers to hospitals registered in the CNES with agreement only to SUS, not listed in the RPS, and no admissions in CIH; “SUS, health plans, and private” refers to those registered in the CNES with agreements with SUS, health plans, or private and included or not in the RPS, with admissions in SIH and CIH, or those registered in the CNES with agreement only with SUS, however included in the RPS or admissions in CIH; and “health plan and private” refers to those registered in the CNES with health plans or private agreements, included or not in the RPS, with no admissions in SIH.

### Multilevel Logistic Regression Model

The multilevel logistic regression model with two levels (patient and hospital) used^21^ is expressed as follows:


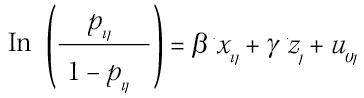


Where: *p*_*ij*_ is the probability of the ith patient of the jth hospital dying during admission; *ß* is the vector of parameters associated with the matrix of covariates of *x*_*ij*_ patients; *γ* is the vector of parameters associated with the variables of the hospital, that is, the second level of the multilevel model; *u*_*0j*_ is the random parameter that measures the variability of outcome between hospital units. It is assumed that *u*_*0j*_ has Normal distribution with zero mean and *σ* ^2^_*ou*_ variance.

The parameters of multilevel models were estimated using MLwiN software, version 2.32. We used the Predictive near-likelihood procedure of second-order, considering the estimation process of MLwiN most suitable for multilevel models of binary response^11^. While standard deviation of the random effect can be directly estimated by the square root of the variance, the calculation of its standard error was obtained by Delta method^7^.

The effects of variables regarding patients and hospitals were interpreted in terms of ratio of chance and their respective 95% confidence intervals. The residual effect of the hospitals was estimated at probabilities of death, calculated according to different levels of random effect: -2 standard deviations below mean, -1 standard deviation below mean, at mean, +1 standard deviation above mean, and +2 standard deviations above mean.

## RESULTS

Of 852,864 hospital admissions selected, 41.0% were due to pneumonia, 30.0% to CHF, 19.0% to CVA, and 10.0% to AMI. The gross mortality rate was 13.5%, with variations of 10.3% (CHF) and 17.3% (CVA) between the selected causes (Table 1).

**Table 1:** Characteristics of the study population. Sao Paulo and Rio Grande do Sul, Brazil, 2008-2010.

Characteristic	Hospital admissions		Deaths	
	
	n	%	n	%
Total	852,864	100.0	115,347	13.5
State of admission				
Sao Paulo	628,596	73.7	91,113	14.5
Rio Grande do Sul	224,268	26.3	24,234	10.8
Sex				
Male	438,443	51.4	58,719	13.4
Female	414,421	48.6	56,628	13.7
Age group				
0-49 years	161,295	18.9	11,239	7.0
50-59 years	133,965	15.7	13,679	10.2
60-69 years	167,902	19.7	20,473	12.2
70-79 years	202,047	23.7	30,474	15.1
80-89 years	152,145	17.8	29,973	19.7
90-99 years	35,510	4.2	9,509	26.8
Main diagnosis				
AMI (I21 Acute myocardial infarction)	85,526	10.0	12,382	14.5
CHF	253,724	29.7	26,254	10.3
I11 Hypertensive heart disease	12,236	1.4	232	1.9
I13 Hypertensive heart and renal disease	763	0.1	29	3.8
I50 Heart failure	240,725	28.2	25,993	10.8
CVA	159,947	18.8	27,708	17.3
I60 Subarachnoid hemorrhage	9,171	1.1	1,956	21.3
I61 Intracerebral hemorrhage	15,715	1.8	4,463	28.4
I62 Other nontraumatic intracranial hemorrhage	5,649	0.7	1,307	23.1
I63 Cerebral infarction	23,286	2.7	3,211	13.8
I64 Cerebrovascular accident, unspecified as hemorrhagic or ischemic	106,126	12.4	16,771	15.8
Pneumonia	353,667	41.5	49,003	13.9
J13 Pneumonia due to *Streptococcus pneumoniae*	662	0.1	66	10.0
J14 Pneumonia due to *Haemophilus infuenzae*	196	0.0	21	10.7
J15 Bacterial pneumonia not classified elsewhere	49,540	5.8	7,097	14.3
J16 Pneumonia due to other specified infectious microorganisms not classified elsewhere	3,155	0.4	358	11.3
J18 Pneumonia by unspecified microorganism	300,114	35.2	41,461	13.8
Comorbidity registered				
No	725,800	85.1	91,665	12.6
Yes	127,064	14.9	23,682	18.6
Charlson Comorbidity Index				
0	822,331	96.4	109,166	13.3
1	23,304	2.7	4,062	17.4
2	7,229	0.8	2,119	29.3
Elixhauser Comorbidity Index				
0	785,586	92.1	104,953	13.4
1	67,278	7.9	10,394	15.4
Use of intensive care unit.				
No	735,324	86.2	80,626	11.0
Yes	117,540	13.8	34,721	29.5
Type of procedure				
Clinical	813,660	95.4	110,010	13.5
Surgical	39,204	4.6	5,337	13.6
Length of stay				
Up to 1 day	66,217	7.8	25,349	38.3
From 2 to 7 days	540,761	63.4	51,566	9.5
From 8 to 14 days	169,913	19.9	22,964	13.5
From 15 to 21 days	51,349	6.0	9,772	19.0
From 22 to 30 days	24,624	2.9	5,696	23.1
Payment of hospital admission				
SUS	645,606	75.7	94,146	14.6
Health plan	178,211	20.9	17,997	10.1
Private	18,706	2.2	1,923	10.3
Philanthropy	10,341	1.2	1,281	12.4
Hospital quality				
Public	241,702	28.3	41,914	17.3
Non-profit private	507,938	59.6	61,831	12.2
For-profit private	103,224	12.1	11,602	11.2
Teaching activity at the hospital				
No	624,546	73.2	82,022	13.1
Yes	228,318	26.8	33,325	14.6
Hospital financing settlement				
Only SUS	119,815	14.0	21,741	18.1
SUS, health plans, and private	637,007	74.7	82,989	13.0
Health plans and private	96,042	11.3	10,617	11.1
Capacity of the hospital				
Up to 49 beds	81,743	9.6	8,040	9.8
From 50 to 149 beds	297,474	34.9	37,815	12.7
From 150 to 299 beds	304,808	35.7	45,894	15.1
300 beds or more	168,839	19.8	23,598	14.0

AMI: acute myocardial infarction; CHF: congestive heart failure; CVA: cerebrovascular accident; SUS: Brazilian Unified Health System

Most admissions were of patients between 60 and 79 years of age. The minority of them possessed some comorbidity, Charlson index other than zero, or presented some Elixhauser comorbidity. Most admissions were for up to seven days. Mostly, the ICU was not used and surgeries were not performed. The payment of hospital admissions was predominantly SUS. Admissions occurred mostly in private hospitals, without teaching activity, capacity greater than 49 beds, and mixed financing settlement: SUS, health plans, and private (Table 1). In hospitals with mixed settlement, admissions paid by SUS were majority. In hospitals of health plans and private settlement, admissions by health plans represented almost the total.

Regarding the risk adjustment model (Table 2), the better capacity of discrimination was from the model 10 (C-statistics = 0.66), which incorporated sex, age group, CCI, Elixhauser comorbidities excluded from CCI, presence of comorbidity, and main diagnosis (Table 2). In the multilevel model (Table 3), higher chances of death were registered in admissions of male patients, in more advanced age groups, with comorbidity, with Charlson index greater than zero, who remained for one day at the hospital, used ICU, performed clinical procedure, and with SUS as source of payment. At the level of hospitals, only the variable capacity proved to be statistically significant, with higher chance of death among larger hospitals (Table 3).

**Table 2:** Risk adjustment models tested. Sao Paulo and Rio Grande do Sul, Brazil, 2008-2010.

Model			C-statistics
Simple models	1	Base model (age and sex)	0.62
	2	Base model + comorbidity	0.63
	3	Base model + CCI	0.62
	4	Base model + Elixhauser index	0.62
	5	Base model + Elixhauser components^a^	0.62
	6	Base model + CCI of main diagnosis	0.62
	7	Base model + main diagnosis	0.65
	8	Base model + main diagnosis group	0.64
Risk adjustment models composed	5	Base model + CCI + comorbidity	0.63
	6	Base model + CCI + Elixhauser index	0.62
	7	Base model + CCI + Elixhauser components^a^	0.62
	9	Base model + CCI + Elixhauser components^a^ + comorbidity	0.63
	10^b^	Base model + CCI + Elixhauser components^a^ + comorbidity + main diagnosis	0.66
	11	Base model + CCI + Elixhauser components^a^ + comorbidity + main diagnosis group	0.65

Source: Brazilian Ministry of Health. National Register of Health Establishments (CNES), Health Plans Registry (RPS), System of Hospital Information of SUS (SIH), and Hospital Admissions Communication (CIH).

CCI: Charlson Comorbidity Index

^a^ Includes other than those referred to in the Charlson Index, significant and with risk effect.

^b^ Final risk adjustment model.

**Table 3:** Multilevel logistic regression model of hospital mortality: ratio of chances of death and estimated confidence intervals. Sao Paulo and Rio Grande do Sul, Brazil, 2008-2010.

Variable	Coefficient	Standard error	Ratio of chance	95%CI
Constant	-3.530	0.117	-	-
First level: patients				
Patient characteristics and risk adjustment				
Sex (ref. cat.: male)				
Female	-0.046	0.007	0.955	0.942-0.968
Age group (ref. cat.: < 50 years)				
50-59 years	0.480	0.015	1.616	1.569–1.664
60-69 years	0.775	0.014	2.171	2.112–2.231
70-79 years	1.141	0.013	3.130	3.051–3.211
80-89 years	1.585	0.013	4.879	4.757–5.005
90-99 years	2.072	0.018	7.941	7.665–8.226
Presence of comorbidity (ref. cat.: no comorbidity)				
With Comorbidity	0.901	0.016	2.462	2.386–2.540
Charlson Comorbidity Index (ref. cat.: CCI = 0)				
CCI = 1	0.211	0.022	1.235	1.183–1.289
CCI ≥ 2	0.726	0.032	2.067	1.941–2.201
Elixhauser components (ref. cat.: no specific comorbidity)				
Cardiac arrhythmia	0.917	0.113	2.502	2.005–3.122
Pulmonary circulation disease	0.776	0.269	2.173	1.282–3.681
Other neurological disease	0.027	0.103	1.027	0.840–1.257
Coagulopathies	0.333	0.147	1.395	1.046–1.861
Weight loss	0.290	0.107	1.336	1.084–1.648
Hydroelectrolytic imbalance	0.144	0.058	0.866	0.773–0.970
Alcohol abuse	-0.300	0.093	0.741	0.617–0.889
Main diagnosis (ref. cat.: I11 hypertensive heart disease)				
I13 Hypertensive heart and renal disease	0.590	0.212	1.804	1.191–2.733
I21 Acute myocardial infarction	1.718	0.073	5.573	4.830–6.431
I50 Heart failure	1.848	0.073	6.347	5.501–7.323
I60 Subarachnoid hemorrhage	2.763	0.078	15.847	13.601–18.465
I61 Intracerebral hemorrhage	3.053	0.075	21.179	18.284–24.533
I62 Other nontraumatic intracranial hemorrhage	2.877	0.081	17.761	15.154–20.817
I63 Cerebral infarction	2.088	0.076	8.069	6.952–9.365
I64 Cerebrovascular accident, unspecified as hemorrhagic or ischemic	2.288	0.073	9.855	8.541–11.371
J13 Pneumonia due to *Streptococcus pneumoniae*	2.139	0.161	8.491	6.193–11.641
J14 Pneumonia due to *Haemophilus infuenzae*	2.391	0.279	10.924	6.323–18.875
J15 Bacterial pneumonia not classified elsewhere	2.499	0.074	12.170	10.527–14.070
J16 Pneumonia due to other specified infectious microorganisms not classified elsewhere	2.180	0.099	8.846	7.286–10.741
J18 Pneumonia by unspecified microorganism	2.454	0.072	11.635	10.103–13.398
Characteristics of the care process and source of payment				
Length of stay (ref. cat.: 1 day)				
2-7 days	-2.152	0.011	0.116	0.114–0.119
8-14 days	-2.112	0.013	0.121	0.118–0.124
15-21 days	1.893	0.016	0.151	0.146–0.155
22-30 days	-1.754	0.020	0.173	0.166–0.180
Use of ICU during hospital admission (ref. cat.: no use)	1.804	0.010	6.074	5.956–6.194
Type of procedure performed (ref. cat.: clinical)	-0.696	0.020	0.499	0.479–0.519
Source of payment of admission (ref. cat.: SUS)				
Health plan	-0.740	0.016	0.477	0.462–0.492
Private	-0.777	0.030	0.460	0.434–0.488
Philanthropy	-0.382	0.037	0.682	0.635–0.734
Second level: hospitals				
Legal nature (ref. cat.: public)				
Non-profit private	-0.255	0.137	0.775	0.592–1.014
For-profit private	-0.163	0.089	0.850	0.714–1.012
Financing settlement (ref. cat.: only SUS)				
Health plans and private	-0.291	0.158	0.748	0.548–1.019
SUS, health plans, and private	-0.214	0.109	0.807	0.652–1.000
Capacity (ref. cat.: < 50 beds)				
50-149 beds	0.331	0.065	1.392	1.226–1.582
150-299 beds	0.277	0.080	1.319	1.128–1.543
300 beds or more	0.373	0.127	1.452	1.132–1.863
Teaching activity (ref. cat.: no teaching is performed)				
Teaching activity is performed	-0.080	0.088	0.923	0.777–1.097
Random effect				
σu	0.685	0.006	1.984	1.960–2.007

ref. cat.: reference category; CCI: Charlson Comorbidity Index; ICU: intensive care unit; SUS: Brazilian Unified Health System

The random effect, regarding the inter-hospital variation unexplained by the variables included in the model, was statistically significant, indicating that the chance of death of patients admitted to hospitals within one standard deviation above the mean e was 98.0% higher than that of patients admitted to hospitals on mean distribution. The Figure shows the probabilities of death for the categories of the variable source of payment according to random effect levels, that depict the variability in mortality between hospitals. Between hospitals within the mean, the greater probability of death was observed in patients with admissions paid by SUS, almost twice of those with health plan or private sources of payment. The probability of death increased significantly among patients admitted to hospitals within two standard deviations above the mean. In the case of patients with hospital admission paid by SUS, the probability of death reached 29.0%. Although admissions financed by health plan or private report less probability to death, this relationship can be changed depending on the hospital in which the patient was admitted to: admissions with health plan as source of payment, if occurred in hospitals within two standard deviations above the mean, had almost twice the probability of admissions whose source of payment was SUS, when admitted to hospitals within the mean (Figure).

**Figure:**
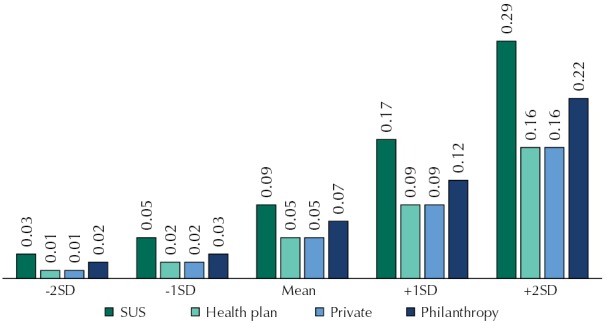
Probability of death per source of payment of hospital admission, according to values of random effects estimated in the multilevel model. Sao Paulo and Rio Grande do Sul, Brazil, 2008-2010.

SD: standard deviation; SUS: Brazilian Unified Health System

## DISCUSSION

In Brazil, few studies analyze the quality of hospital care measured by risk-adjusted mortality employing multilevel models. Yet, the results found align to those of national studies identified^16,20^. At the international level, if on one hand the selection of four of the seven groups of causes recommended by AHRQ brings the need for adaptations for comparison between publications with similar approaches, on the other hand, there is variability in the set of selected code in European studies, corroborating the need of local adaptations as the one carried out^5,19^.

Regarding risk adjustment, the variables here used have similarities with those used in international and national studies, for their recognized relationship with the risk of death and availability in databases^1,6,12,23,25,27^. On the other hand, in relation to information about comorbidity and related indexes, although less used in Brazilian studies because of the incompleteness of the records^16^, they were included in this study aiming at improving the predictive ability of risk adjustment model and, above all, the conceptual coherence with the analytical approach.

The treatment applied to length of stay and some of the results were similar to those studies that use global approach of hospital mortality^14,17^. The high risk of death observed on the first day of admission is possibly related to emergency cases, especially those requiring palliative care or with lower therapeutic possibilities^17^. On the other hand, the global approach of hospital mortality uses the length of stay as adjustment variable^14^, option here excluded, since the length of stay may express greater gravity, adverse events resulting from problems in the quality of care or availability of beds for long term care^15^.

Regarding the association between hospital mortality and hospital characteristics, we expected to find a lower risk of death in larger hospitals, for their better structure and for the relationship between volume and quality described in literature^3^. However, the analysis showed a higher risk in larger hospitals, similar to what Garcia et al.^12^ observed. In addition, the gradient of risk of death was similar between capacity classes, differentiating only hospitals with capacity smaller or greater than 50 beds. Despite the minimum cut of hospital volume in one case a day, smaller hospitals seem to present specific role with some degree of experience in hospital care of the causes studied, factor that may contribute to the better outcome of care.

The analysis showed the effect of the source of payment on the risk of death. Patients with private health plan or that pay out their own pocket (private) showed lower adjusted mortality rates than patients of SUS, although admitted to the same hospitals. Similar results were published in international studies, mainly from the USA, where the benefits are of patients covered by private insurances, when compared with those covered by public insurances^25,27^. In Brazil, Martins et al.^15^ also found higher risk of death among patients of SUS, however, they did not studied possible sources of payment disparities within the same hospitals, here explored by the analysis of financing settlements.

Studies conducted in the United States showed higher risk of death among uninsured patients and with private payment^8,25^. This difference may be related to different eligibility of patients to public health services in both countries. In Brazil, the entire population is eligible to SUS; in the USA, some people are ineligible for public insurance and also do not have private insurance. Thus, while private patients in the USA are mostly excluded from both modalities (public and private), in Brazil patients who pay private hospitalization tend to have higher purchasing power or are beneficiaries of the best health plans, with good margins of reimbursement.

Of the factors that contribute to disparities in the risk of death among sources of payment we highlight the differences between clinical practice, access to technology, and procedures of high cost and complexity^8^. Analyzing the Brazilian health system, Victora et al.^26^ suggests that service providers that attend SUS patients and non-SUS patients offer differentiated standard of care according to the values they receive, which would influence the choice of procedure and material used, thus affecting quality of care and possibly the risk of death or occurrence of other adverse results.

Although there were privileged variables in this study, the effect of the financing settlement and legal nature of the hospital on the adjusted mortality showed no significance. However, although this feature had not been able to differentiate the risk of death in hospitals, we observe, within the same physical structures, differences between patients with source of payment SUS and non-SUS. This suggests that even physically available in hospitals, some resources are not available to patients of SUS, indicating iniquities in the process of hospital care.

Some limitations are inherent to the use of administrative databases, often designed originally to billing services. Therefore, their content may not comprise the whole set of information necessary for analyses of quality of services, or there may be incomplete or incorrect data collection, affecting the analyses developed. Nevertheless, the use of such data is an alternative explored in many countries due to the ease of obtaining, comprehensiveness, and continuity^15,a^. This study highlights the use of CIH, which, despite representing the only source about non-SUS admissions in the country, is little used in Brazilian studies, often due to its questionable quality. Considering the variables of interest and the need to use the CIH to the proposed analyses, we opted for the delimitation of the study universe in two states with best coverage, in which we also observed reasonable degree of consistency in the data, according to a study of Machado^a^.

Incompleteness of data on the severity profile of patients in Brazilian information systems, both public and private, outstands as another limitation. Incompleteness has been discussed in evaluation studies, since it prevents more accurate risk adjustments, compromising the accuracy of the analysis. In this sense, higher risk of death in public hospitals observed in this study may reflect failures in risk adjustment for not precisely measuring the greater severity of patients who use public services, possibly those with the worst socioeconomic conditions. However, the use of the source of payment as indicative of the health condition of the patient at the time of admission would not be an appropriate solution, since it would exclude from the analysis problems in the quality of care provided associated with discrimination or inequity. A specific restraint was the noninclusion of the risk model of the type of admission (elective or emergency), due to its nonfulfillment at the base of CIH, since national studies found relationship with the chance of death^12,20^.

The restricted degree of some categorical variables brought difficulties and also represents a limitation in the analyses. Especially in the case of financing settlements, each of the three categories studied includes groups of hospitals with varied proportion of sources of payment of admissions that compose its clientele. Are distinct hospitals that attend 20.0% or 80.0% of the patients by SUS, and this difference possibly influenced in varying degrees the result of the care, especially considering that sources of showed significant effect on the risk of death.

Although presenting limits that point to the need for improvement and expansion of clinical information registered in databases, the approach used in this study to assess hospital mortality, with the application of risk adjustment, multilevel regression risk, and use of administrative data, allowed more accurate estimation of the effect of the characteristics of admissions and hospitals on the risk of death. Even after controlling the effects of individual risk and considering the hierarchy between the levels of analysis, the findings of this study indicate the existence of differences in the quality of hospital care, measured by adjusted mortality, according to sources of payment of the hospital admission. This analysis indicated a disadvantage for SUS patients when compared with patients of health plans or with private payment, including when admitted to the same hospital. In addition to structural characteristics of the hospitals and the profile of the patients, the elaboration of policies aimed at this area should also consider the coverage of the population by health plans, the network shared between beneficiaries of plans and users of SUS, the dynamics of attendance to the various sources of payment by hospitals and, most importantly, how these factors influence the clinical performance. Therefore, they would contribute to the reduction of inequalities and to improve the effectiveness of the health system.

## Footnotes

a Machado JP. O arranjo público-privado no Brasil e a qualidade da assistência hospitalar em São Paulo e no Rio Grande do Sul [these]. Rio de Janeiro (RJ): Escola Nacional de Saúde Pública, Fundação Oswaldo Cruz; 2014.

b Department of Health and Human Services (UEA), Agency for Healthcare Research and Quality. Guide to inpatient quality indicators: quality of care in hospitals: volume, mortality, and utilization. Version 3.1, Rockville, MD: Agency for Healthcare Research and Quality; 2007.
